# Willingness to pay for health care services in common cold, retinal detachment, and myocardiac infarction: an internet survey in Japan

**DOI:** 10.1186/1472-6963-6-12

**Published:** 2006-02-20

**Authors:** Hideo Yasunaga, Hiroo Ide, Tomoaki Imamura, Kazuhiko Ohe

**Affiliations:** 1 Department of Planning, Information and Management, University of Tokyo Hospital, Tokyo, Japan

## Abstract

**Background:**

The application of Willingness To Pay (WTP) measurement with Contingent Valuation Method (CVM) to medical services is gradually increasing. Knowing what influences WTP is an important matter because validity of CVM in medical services remains controversial. The objective of this survey is to measure WTP for the treatment of typical acute illnesses and to analyze the factors affecting WTP.

**Methods:**

A questionnaire survey was conducted over the Internet, in which 795 men and women between 40 and 59 years old responded to questions about WTP for medical expenses in three hypothetical scenarios: common cold (CC), retinal detachment (RD) and myocardiac infarction (MI).

**Results:**

Mean WTP was $29.9 for CC, $2,233 for RD, and $8,976 for MI. WTP for RD and MI was lower in the low-income group. While WTP for CC did not vary with income, WTP was higher in groups whose current subjective fitness levels were low.

**Conclusion:**

Although WTP measurements are criticized frequently for their validity and reliability, they are still useful for determining the economic value of medical services. Based on the results of this study, it is deemed necessary to enhance safety nets for low-income earners in regards to serious illnesses that incur high medical expenses. Further, it is recommended that the rate of co-payments be set relatively high with respect to mild illnesses for which alternative services are available.

## Background

In ordinary markets, consumers compare and examine quality and price when making a purchasing decision on goods and services. Normally, prices of goods and services are clearly indicated to consumers. This, however, is not the case for medical services.

In many developed and developing countries, medical care is regarded as one of the public services. In the 1960s, Japan introduced the universal health insurance system, enabling patients to receive most medical services by bearing only part of the expenses. The health insurance system in Japan primarily adopts a complete fee-for-service scheme, and an official price is set with respect to each individual medical act in detail. However, information on the official price of medical care is hardly disclosed to the Japanese people. Further, as the onset of illness is unpredictable in most cases and the opportunity to demand medical services always arises incidentally, it is almost impossible for patients to collect information on the price of services in advance. It is therefore difficult for individuals to evaluate the price when purchasing medical services, in contrast with the purchase of ordinary goods and services.

Is it possible to estimate the fair price of medical services from the consumers' point of view? The most basic method is to measure the Willingness to Pay (WTP) for medical services. WTP is frequently used as a benefit in cost-benefit analysis, and is used widely in the field of medical economics. Additionally, its convenience and limits have already been clarified [1-3]. On the other hand, WTP can be used for the purpose of acquiring information on fair pricing and demand forecast, from the viewpoint of medical marketing [4,5]. A number of preceding studies have measured WTP for medical services for such purposes [6,7].

Contingent Valuation Method (CVM) is a method developed for actual WTP measurement [8,9]. It originally aimed at measuring the benefits of services that have no market, such as environmental measures. It can, however, also be applied to public services that are not traded through the market mechanism in free economies. WTP has been measured for various medical services, ranging from prevention to treatment [10].

This paper measures WTP for medical services, by presenting hypothetical scenarios for a number of acute illnesses. It then attempts to estimate the respective prices of medical services deemed reasonable by general public. It also analyzes the factors that affect WTP for medical services.

## Methods

### Subject

A survey was conducted on WTP for medical services, based on a questionnaire survey using the Internet. The population was assumed to be general public between 40 and 59 years old residing in Japan. For the survey, cooperation was obtained from an Internet research company located in Tokyo, with approximately 195,000 Internet users registered as monitors. 2,500 were randomly selected from the registered men and women between 40 and 59 years old (approximately 52,200 people). Emails requesting cooperation in the questionnaire survey were sent all at once at 19:00, February 22, 2005. Individuals subject to the survey were able to respond and reply to the questionnaire voluntarily and anonymously by accessing the website where the questionnaire survey was conducted, and by entering the answers directly on the survey form on the web.

This research is simply based on the anonymous self-administered questionnaire without experimental interventions. From the cover letter of the questionnaire, the subjects were well informed that 1) data collection and analysis was fully anonymous so that their private information would be completely protected, 2) all the answers would be kept confidential, processed statistically, and used only for scientific study, and 3) they could either participate of their own accord or refuse to participate.

We aimed at collecting 700 to 800 samples. Since the sample size was not based upon any pre hoc power calculations, this was a sample of convenience. Each time after e-mail transmission, the number of response was checked. 795 responses were collected in only 48 hours. At that point, we immediately closed down the website and terminated the survey. The response rate was calculated as 31.8% (= 795/2500). All questions in the survey form were multiple-choice questions, and the form was configured in a way that made it impossible to move on to the next question if a question was left unanswered. This made it possible to acquire completely valid responses with no missing data in all samples, and inevitably resulted in a valid response rate of 100%.

### Hypothetical scenarios for asking WTP for medical services

Acute illnesses with subjective symptoms were first divided into the following three categories:

Category 1: There is no risk of death or disability.

Category 2: There is no risk of death, but there is a risk of serious disability.

Category 3: There is a risk of death.

Common cold (CC), retinal detachment (RD) and acute myocardiac infarction (MI) were chosen as illnesses that fall into Categories 1, 2 and 3, respectively. Hypothetical scenarios relating to these three illnesses were presented, and the WTP for medical services was asked. This made it possible to estimate the respective prices of individual medical services deemed reasonable by general public, with given assumptions on symptoms and conditions. Individuals subject to the survey were required to select one of the prices from among the seven options presented in each question, based on "payment cards" [11-13]. The hypothetical scenarios and the options presented were as follows.

(i) Category 1 (Common Cold, CC)

Question: "Suppose you have a temperature of 38.5 degrees Celsius, feel lethargic from head to toe and visit a medical institution as an outpatient. You are diagnosed with a common cold, and are prescribed oral medicine. What is the maximum price you are willing to pay for the medical services provided?"

Options: $10, $20, $30, $40, $50, $100, and $200

(ii) Category 2 (Retinal Detachment, RD)

Question: "Suppose you are at risk of losing sight in one of your eyes because your left eye suddenly suffers retinal detachment. As a result of surgery performed on your left eye and twelve days of hospitalization, you avoid becoming blind. What is the maximum price you are willing to pay for the medical services provided?"

Options: $1,000, $1,500, $2,000, $2,500, $5,000, $10,000, and $20,000

(iii) Category 3 (Myocardiac Infarction, MI)

Question: Suppose your life is at risk as you suddenly suffer acute myocardiac infarction, and you are taken to hospital by ambulance without regaining consciousness. You escape death as a result of receiving cardiopulmonary resuscitation and undergoing emergency percutaneous coronary intervention. After two months of hospital treatment, you more or less restore your health and are discharged from hospital. What is the maximum price you are willing to pay for the medical services provided?"

Options: $3,000, $6,000, $9,000, $15,000, $30,000, $60,000, and $120,000

### Basic attributes of individuals subject to survey

The survey included questions on factors deemed to affect WTP such as age, sex, annual household income, private health insurance policy holding status, hospitalization history, and subjective fitness level.

In the question on private health insurance policy holding status, three options were given: "Have insurance", "Don't have insurance", and "Don't want to answer". As Japan implements a national health insurance plan, almost all Japanese citizens can reap the benefits of public health insurance. If a patient receives medical services based on public health insurance, he/she must incur co-payments accounting for a certain percentage of the expenses. Such percentage is prescribed by law (such as the Health Insurance Law), uniformly at 30%, for all standard medical services covered by insurance, excluding elderly persons and children. To prepare for the 30% co-payment burden, many Japanese people buy private medical insurance services sold by private insurance companies. However, the actual number of buyers of such services across Japan is unknown.

As for the subjective fitness levels, the individuals were asked to describe their current status on five levels: "Very good", "Good", "Average", "Slightly bad" and "Bad".

### Compilation of WTP data

Mean WTP and median WTP were calculated with respect to each scenario. Variable *X *denoted each presented price, while the number of individuals who are willing to pay more than the presented price (i.e., individuals who can tolerate that price) denoted *the number of tolerant individuals *(*Y*). The values were plotted on an *X*-*Y *plane and approximated on the basis of the following exponential function.

*Y *= *αe*^*βX *^(*)

*X*: Price, *Y*: Number of tolerant individuals, *α*, *β*: Constants

Constants *α*, *β *in the formula above (*) and the coefficient of determination adjusted for the degrees of freedom *R*^2 ^were calculated.

### WTP by annual income

The relationship between an individual's WTP and his/her income was studied.

Firstly, the sample group was divided into three groups: low-income group with an annual income less than 40,000 US dollars (92 individuals), medium-income group with an annual income of 40,000 US dollars or more but less than 80,000 US dollars (371 individuals), and a high-income group with an annual income of 80,000 US dollars or more (266 individuals). In total, 729 individuals were subject to analysis, excluding 66 individuals who responded "Don't want to answer" the question on annual income. With respect to each scenario and group, presented prices was plotted on the horizontal axis, while the ratio of individuals who can tolerate purchasing the medical services at a higher price than the presented price was plotted on the vertical axis. This made it possible to visually represent how WTP varied with the income level.

Next, the mean WTP was compared among the three groups (low-income, medium-income and high-income groups) in each scenario. Due to doubts about the normality and homogeneity of variance regarding the distribution of WTP, a Kruskal Wallis test (nonparametric test) was adopted.

Further, in order to adjust various factors at the same time, regression analysis was conducted assuming that WTP is a dependent variable and that annual income, age, sex, private health insurance policy holding status, hospitalization history, and subjective fitness level are independent variables. Taking into account that the dependent variable is ordinal data, ordinal regression analysis was adopted rather than normal multiple regression analysis. Pseudo-r squares were calculated for the ordinal regression models [14].

P values of less than 0.05 were considered to be statistically significant. All statistical analyses were performed using statistics software SPSS ver.13.0 (SPSS Ltd., Chicago, USA). The exchange rate is assumed to be 105 yen for the U.S. dollar.

## Results

### Descriptive statistics (Table 1)

The mean age was 46.2 ± 4.9 years old. As for age distribution, the percentage decreased as the age increased. The mean annual income was $75,080 ± 34,190. The subtotal of individuals with an annual income of less than $40,000 accounted for 12% of the total. There were 371 individuals earning between $40,000 and $80,000 (47%), and 266 individuals earning $80,000 or more (34%).

Those who answered "Have private insurance" accounted for 68% of the total. In regards to the question "Have you ever been hospitalized?", 381 individuals answered "Yes" (48%). When asked about their subjective fitness levels, 223 individuals answered "Very good" or "Good" (28%), 376 answered "Average" (47%), 196 answered "Slightly bad" or "Bad" (25%).

### Compilation of WTP data

(i) Category 1 (CC)

Mean WTP for medical services for CC was $29.9, while median WTP was $30. As for the distribution of WTP, 44, 256, 336, 67, 77, 14 and 1 individuals had a WTP of $10, $20, $30, $40, $50, $100 and $200, respectively.

(ii) Category 2 (RD)

Mean WTP for medical services for RD was $2,233, while median WTP was $1,500. As for the distribution of WTP, 248, 166, 209, 66, 85, 15 and 6 individuals had a WTP of $1,000, $1,500, $2,000, $2,500, $5,000, $10,000 and $20,000, respectively.

(iii) Category 3 (MI)

Mean WTP for medical services for MI was $8,976, while median WTP was $6,000. As for the distribution of WTP, 250, 230, 149, 115, 44, 6 and 1 individuals had a WTP of $3,000, $6,000, $9,000, $15,000, $30,000, $60,000 and $120,000, respectively.

**Figure 1:**
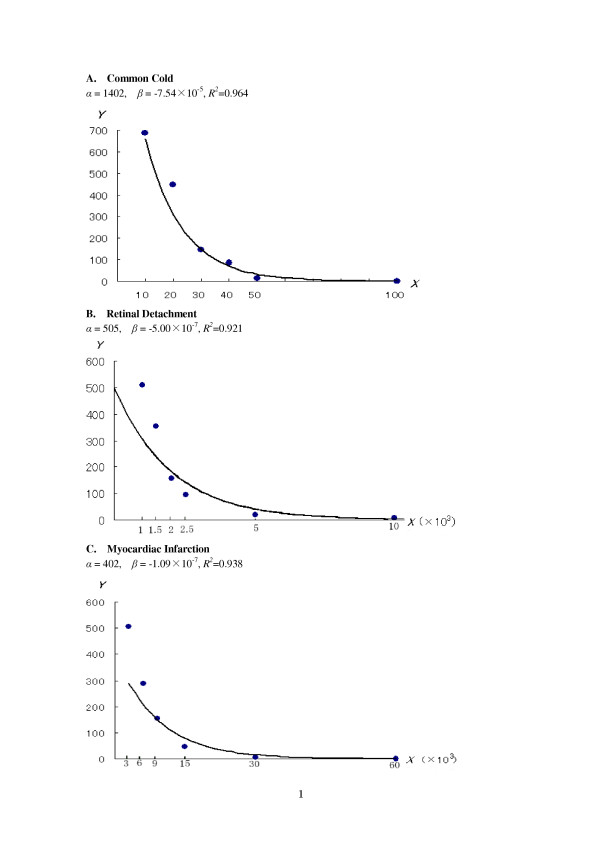
**The relationship between the presented price and the number of individuals who are willing to pay more than the price**. *Y *= *α e*^*βX*^, *X*: presented price [USD], *Y*: the number of tolerant individuals, *α*, *β*: constant, *R*^2^: coefficient of determination.

A figure of the function of the price and the number of tolerant individuals was also depicted with respect to each scenario. (Figure 1)

### WTP by annual income

**Figure 2:**
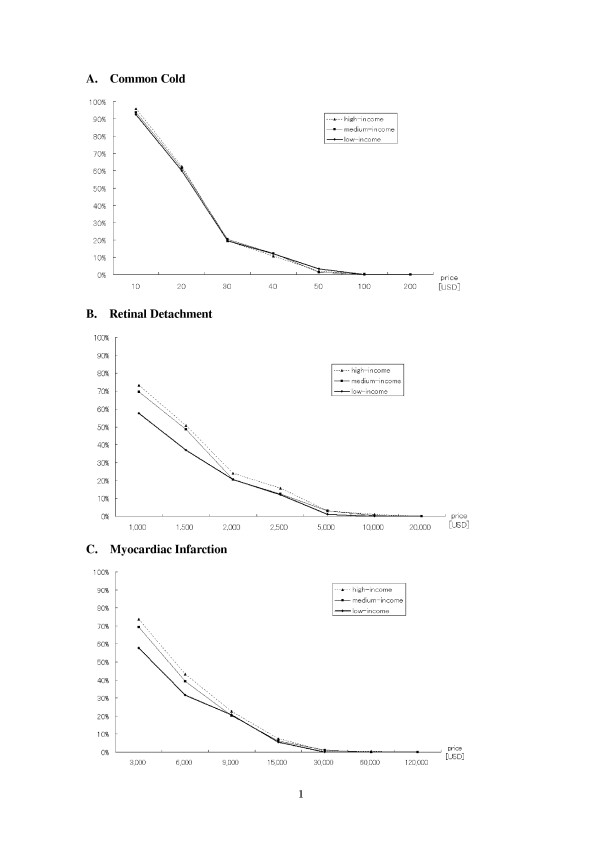
**WTP by Annual Income**. Horizontal axis: presented price, Vertical axis: the ratio of individuals who can tolerate purchasing the medical services at a higher price than the presented price.

The sample group was divided into three groups by annual income, and a graph was depicted with respect to each group, in which presented price was plotted on the horizontal axis and the ratio of individuals who can tolerate purchasing the medical services at a higher price than the presented price was plotted on the vertical axis. In the case of CC (Figure 2-A), the three graphs almost overlapped. In the case of RD (Figure 2-B) and MI (Figure 2-C), the low-income group with an annual income of less than $40,000 was skewed downwards compared to the other two groups.

Table 2 shows the mean WTP in each scenario and group. According to the results of the nonparametric test, in which the respective mean values of WTP of the three groups were compared with respect to each of the three scenarios, a significant difference was found among the three groups in the case of RD (p = 0.044) and MI (p = 0.049), and the WTP was significantly low in the low income group.

Further, in order to adjust the impact of various factors, an ordinal regression analysis was conducted. WTP relating to CC was reorganized into four categories ($10, $20, $30 and $40 or more), as were WTP relating to RD ($1,000, $1,500, $2,000 and $2,500 or more) and WTP relating to MI ($3,000, $6,000, $9,000 and $15,000 or more). As for the subjective fitness level, "Very good" and "Good" were merged, as were "Bad" and "Slightly bad", to be consolidated into three categories ("Good", "Average" and "Bad").

Low-income was not a significant factor that affects WTP in the case of CC (p = 0.574). WTP was significantly higher in the "Average" and "Bad" groups than the "Good" group with respect to subjective fitness level (p = 0.015 and 0.026, respectively). (Table 3-A) As for factors affecting WTP in the case of RD and MI, the low-income group was significant (p = 0.003 and 0.003, respectively). That is to say, the WTP of the low-income group was significantly lower than in other groups. (Table 3-B, C).

Further, the results indicated that the WTP in the case of MI was significantly lower among men than women (p = 0.023). (Table 3-C)

## Discussion

### Advantages and limitations of questionnaire surveys using the Internet

While there are established methods of conducting questionnaire surveys, such as mail surveys, interview-based surveys, group surveys and phone surveys, the use of Internet surveys has been spreading in recent years. They are frequently used by commercial enterprises for market research, but few have been applied to academic research.

Drawbacks of Internet surveys are that the Internet users are relatively skewed towards the young generation, and that sampling errors occur because respondents are limited to active Internet users [15]. In this study, individuals subject to the survey were limited to people between 40 and 59 years old, in order to prevent the sample group from being skewed towards the young generation.

In Japan, Internet users have been increasing rapidly in recent years. In 2003, the Internet penetration rate reached 88% of all households. The penetration rate among individuals in twenties and thirties – which was only 6% and 7% in 1996 – exceeded 90% in 2003. The penetration rate among individuals in forties or fifties – which was merely 4% and 3% in 1996 – also increased dramatically, reaching 85% and 63% in 2003 [16]. The problem of users being skewed towards the young generation can be solved eventually, along with the explosive dissemination of the Internet.

However, it can be thought that there are not many active users of the Internet who register as monitors with research companies and respond to questionnaires. Samples obtained by the sampling of only voluntary applicants tend to give rise to sampling errors compared to samples obtained through random sampling. This problem has not been solved in this study.

On the other hand, the Internet surveys have several advantages, including the ability to collect large volumes of data in a very short period of time, the ability to substantially reduce the data processing workload and the relatively low costs [15]. Another major advantage is the ability to avoid invalid responses.

In this study, relatively low response rate 31.8% was obtained. This may have induced self-selection bias, i.e. bias by the difference of characteristics between those who participated in research spontaneously and those who did not. Participants may have had a stronger concern about health or felt the burden of medical expenses more strongly than non-participants.

### WTP for medical expenses

Questions on WTP were asked in three hypothetical scenarios, based on the payment cards method.

Hypothetical scenarios in this research posed absolute outcomes. Indeed, there are various relative outcomes that are more realistic. However, it is not appropriate to incorporate various risks in a short scenario, because a complicated scenario creates a bias triggered by the subjects' misunderstanding of the content.

The number of individuals who can tolerate a price higher than the presented price (*X*) was defined as *the number of tolerant individuals *(*Y*). The number of tolerant individuals (*Y*) differs from the "demand" at that price. This is because even if the price is higher than what the individual can tolerate, he/she still has to bear the burden due to the lack of alternative services to many medical services. In the case of illnesses such as common cold (people are familiar with how to treat them and alternative services are available, including over-the-counter medicine and folk medicine), the number of tolerant individuals (*Y*) may be deemed to be an approximation of demand. In the case of CC, the mean WTP for initial outpatient consultation was $29.9. If the actual amount of co-payment exceeds the tolerable limit, the individual is expected not to go for outpatient consultation.

In contrast, there are no alternative services available in the remaining two scenarios (RD and MI), and the failure to purchase the medical services may lead to permanent disability or death. Therefore, an individual rarely decides not to purchase the services even if the actual amount of co-payment exceeds his/her tolerable limit. In other words, a huge disparity is deemed to exist between the number of tolerant individuals (*Y*) and the actual demand.

The validity of WTP obtained in this study need to be examined in a number of aspects. In the case of surgery performed to avert blindness due to RD, the mean WTP for hospitalization expenses was $2,233. This accounts for only about 3% of the average annual income ($75,080). In the case of cardiopulmonary resuscitation and cardiac catheter intervention performed to avert death from MI, the mean WTP for hospitalization expenses was $8,976. This accounts for only about 12% of the average annual income. However, such WTP cannot be equated with "the value of one eye" or "the value of life". The WTP presented in a hypothetical situation differs from the actual amount paid – a typical criticism against CVM [4].

Another risk is the so-called "strategic bias" [8,9]. This refers to the tendency of individuals subject to surveys to avoid giving responses that are detrimental to them. As nationwide compulsory health insurance is completely accepted in Japan, citizens who believe that the costs of medical services should be borne by the public sector might have a psychological aversion to bearing high medical expenses.

There are various elicitation methods for CVM such as open-ended questions, payment cards, bidding games, dichotomous choices, and so forth. Under the open-ended questions (direct questions), the respondents are invited to their own WTP valuation, unbounded and unprompted. Although it is the simplest method, it imposes a large task on the respondents and is likely to produce a large number of non-responses [1,4,9,10]. This problem may be overcome by presenting options of possible answers to the respondents. The payment cards method was selected in this study. In this method, the respondents were presented with various amounts of money and requested to choose their own WTP from them. Payment cards method have a unique advantage: it matches the purchasing behavior of "shopping around" (i.e., individuals visit a number of stores that sell the same goods and services at different prices) [12,13]. Attention must be paid to the existence of the so-called "range bias" in the payment cards method [8,9]. This means that the WTP responses are affected by the range of the presented amounts. In this study, WTP responses for RD and MI were a little maldistributed in the lower amounts. This finding suggests that the range of the presented amounts was somewhat broad.

As for the bidding game, it underlies the risk of a starting point bias. The respondents are asked whether they are willing to pay the first given amount. The bid is raised or lowered depending on their answers like an auction process. With this method, the maximum WTP is influenced by the first bid [9,17,18].

### WTP by annual income

The impact of income on WTP has been clearly shown theoretically in preceding studies [5]. Empirical studies that measured WTP with respect to ischemic heart disease have also revealed that WTP is relatively low among low-income earners [19,20]. In this study, no significant differences were found among the low-income, medium-income and high-income groups in terms of WTP for medical expenses for CC. WTP for medical expenses for RD and MI was found to be significantly lower in the low-income group.

What are the policy implications of the aforementioned results?

The fact that WTP with respect to serious illnesses varies with income level implies the need to enhance safety nets for the low-income group. In other words, it is necessary to take measures to reduce the burden of low-income earners in regards to serious illnesses which incur high medical expenses. Japan has a benefits scheme for high medical expenses: if the monthly co-payment for inpatient expenses exceeds a certain amount, the excess amount is reimbursed. The monthly co-payment limit varies with income. The limit for patients under the age of seventy is normally ¥72,300 (= $689) or more and the limit for high-income earners is ¥139,800 (= $1,331) or more, whereas the limit for low-income earners is ¥35,400 (= $337). This policy is in harmony with the implications of this study.

In this study, WTP for medical expenses for CC did not vary with income. The same may apply to other mild illnesses with the possibility of healing naturally, or for which alternative services are available, such as over-the-counter medicine. In Japan, patients are guaranteed free access to all medical institutions. General practitioners provide primary care, and hospitals provide not only inpatient care but also outpatient care that widely range from primary care to secondary care. Even patients with the common cold can freely visit any medical institution as new patients in principle.

The annual average frequency of outpatient consultation per patient is 5.8 times in the United States, 6.1 times in Great Britain, 6.5 times in Germany, and 6.5 times in France, compared to 16.0 times in Japan, which is exceptionally high [21]. Many of them are deemed to involve consultation for non-critical illnesses, including consultation for mild illnesses such as common cold.

In Japan, a 30% co-payment burden is currently imposed across the board, regardless of the type and severity of the illness. There is much room for improvement in this area, and it is recommended that the co-payment rate for mild illnesses for which alternative services are available be set higher than the co-payment rate for serious illnesses. For instance, in France, the co-payment rate for general medical treatment by a general practitioner is higher than the rate for serious illnesses [22]. Such policy is not inconsistent with the implications of our study; a higher co-payment rate may restrain trivial visits. This may limit the frequency of outpatient consultation and mitigate the burden of medical expenses incurred by the public sector. However, our conclusion about co-payments for trivial visits is applicable only to the nations where free access to medical institutions is guaranteed and cannot be applied in the nations where access to medical institutions is restricted and the number of primary care visits is low.

### Other factors affecting WTP

As for the subjective fitness level, WTP was significantly higher in the "Average" and "Bad" groups than the "Good" group in the case of the common cold. In the other two cases, subjective fitness level did not significantly affect WTP. It is difficult to interpret this finding strictly. Possibly, some people of "Good" subjective fitness level may be overconfident of their health. Because the common cold often spontaneously improves, they do not particularly want to pay money for medical institutional services to recover from the common cold.

Most of the variables in our study are ordinal or categorical data based on a questionnaire survey. The pseudo-r squares in Table 3 are relatively low and the fitness of the regression equations is not so good. This finding may suggest the existence of an unknown variable affecting WTP.

## Conclusion

A questionnaire survey was conducted over the Internet, in which 795 men and women between 40 and 59 years old responded to questions about WTP regarding medical expenses in three hypothetical scenarios. Mean WTP was $29.9 for CC, $2,233 for RD, and $8,976 for MI. WTP for RD and MI was lower in the low-income group, while WTP for CC did not vary with income. Although WTP measurements are criticized frequently for their validity and reliability, they are still useful for estimating the economic value of medical services. The results of this study indicate the need to enhance safety nets for low-income earners in regards to serious illnesses that incur high medical expenses. Further, it is recommended that the co-payment rate be set relatively high for mild illnesses for which alternative services are available.

## Competing interests

The author(s) declare that they have no competing interests.

## Authors' contributions

HY, HI, TI and KO jointly conceived the idea for the study. HY analyzed the data and all authors interpreted the results. HY drafted the manuscript. All authors revised the paper and approved the final version. All authors take public responsibility for appropriate portions of the content of the manuscript.

## Pre-publication history

The pre-publication history for this paper can be accessed here:



## Figures and Tables

**Table 1:** Descriptive Statistics (n = 795)

**Parameter**	**Value**	**%**
**Age**		
40–44	385	48%
45–49	218	27%
50–54	125	16%
55–59	67	8%
**Sex**		
Male	463	58%
**Annual income**		
<10,000 USD	13	2%
10,000–20,000	11	1%
20,000–30,000	27	3%
30,000–40,000	41	5%
40,000–60,000	153	19%
60,000–80,000	218	27%
80,000–100,000	139	17%
100,000–150,000	105	13%
>150,000	22	3%
Don't want to answer	68	8%
**Private insurance policy holding status**		
Have Insurance	546	69%
Don't have insurance	221	28%
Don't want to answer	28	4%
**History of hospitalization**		
Yes	381	48%
**Subjective fitness level**		
Very good	58	7%
Good	165	21%
Average	376	47%
Slightly bad	178	22%
Bad	18	2%

**Table 2:** Mean WTP by annual income and results of Kruskal Wallis test for comparison of mean WTP among three groups

Annual Income	Common Cold	Retinal Detachment	Myocardiac Infarction
Low income group (n = 92)	30.0	1,929	7,728
Medium income group (n = 371)	29.7	2,205	8,725
High income group (n = 266)	29.8	2,399	9,440

p-value	0.910	0.044	0.049

**Table 3:** Results of ordinal regression analysis

**A. Common Cold (n = 729)**			
**Parameter**	**B-coefficient**	**Wald**	**p-value**

**Age**			
40–44	-0.118	0.58	0.447
45–49	0.017	0.01	0.917
50–54	0.075	0.19	0.667
55–59	Reference		
**Sex**			
Male	-0.016	0.03	0.853
Female	Reference		
**Annual income**			
Low	-0.077	0.32	0.574
Medium	0.014	0.02	0.876
High	Reference		
**Private insurance policy holding status**			
Have insurance	0.058	0.40	0.527
Don't have insurance	Reference		
**History of hospitalization**			
Yes	0.075	0.83	0.362
No	Reference		
**Subjective fitness level**			
Bad	0.277	5.96	0.015
Average	0.218	4.97	0.026
Good	Reference		
Pseudo-r square = 0.018			

**B. Retinal Detachment (n = 729)**			

**Parameter**	**B-coefficient**	**Wald**	**p-value**

**Age**			
40–44	-0.011	0.00	0.949
45–49	-0.099	0.30	0.586
50–54	-0.023	0.01	0.904
55–59	Reference		
**Sex**			
Male	-0.077	0.67	0.414
Female	Reference		
**Annual income**			
Low	-0.484	8.98	0.003
Medium	-0.136	1.86	0.172
High	Reference		
**Private insurance policy holding status**			
Have insurance	-0.012	0.01	0.905
Don't have insurance	Reference		
**History of hospitalization**			
Yes	0.172	3.48	0.062
No	Reference		
**Subjective fitness level**			
Bad	0.184	2.07	0.150
Average	0.142	1.62	0.203
Good	Reference		
Pseudo-r square = 0.021			

**C. Myocardiac Infarction (n = 729)**			

**Parameter**	**B-coefficient**	**Wald**	**p-value**

**Age**			
40–44	0.117	0.42	0.518
45–49	0.099	0.28	0.597
50–54	0.177	0.78	0.377
55–59	Reference		
**Sex**			
Male	-0.215	5.18	0.023
Female	Reference		
**Annual income**			
Low	-0.481	8.82	0.003
Medium	-0.157	2.47	0.116
High	Reference		
**Private insurance policy holding status**			
Have insurance	-0.004	0.00	0.969
Don't have insurance	Reference		
**History of hospitalization**			
Yes	0.064	0.48	0.490
No	Reference		
**Subjective fitness level**			
Bad	0.087	0.46	0.496
Average	0.104	0.88	0.349
Good	Reference		
Pseudo-r square = 0.022			
